# Mental Health Practitioners’ Immediate Practical Response During the COVID-19 Pandemic: Observational Questionnaire Study

**DOI:** 10.2196/21237

**Published:** 2020-10-01

**Authors:** Shannon E Reilly, Katherine L Zane, William T McCuddy, Zachary A Soulliard, David M Scarisbrick, Liv E Miller, James J Mahoney III

**Affiliations:** 1 Department of Behavioral Medicine and Psychiatry West Virginia University Morgantown, WV United States; 2 Department of Neuroscience West Virginia University Morgantown, WV United States; 3 Rockefeller Neuroscience Institute West Virginia University Morgantown, WV United States

**Keywords:** COVID-19, clinical practice, tele–mental health, mental health, survey

## Abstract

**Background:**

The COVID-19 pandemic has been associated with increased psychological distress, signaling the need for increased mental health services in the context of stay-at-home policies.

**Objective:**

This study aims to characterize how mental health practitioners have changed their practices during the pandemic. The authors hypothesize that mental health practitioners would increase tele–mental health services and that certain provider types would be better able to adapt to tele–mental health than others.

**Methods:**

The study surveyed 903 practitioners, primarily psychologists/doctoral-level (Psych/DL) providers, social workers/master’s-level (SW/ML) providers, and neuropsychologists employed in academic medical centers or private practices. Differences among providers were examined using Bonferroni-adjusted chi-square tests and one-way Bonferroni-adjusted analyses of covariance.

**Results:**

The majority of the 903 mental health practitioners surveyed rapidly adjusted their practices, predominantly by shifting to tele–mental health appointments (n=729, 80.82%). Whereas 80.44% (n=625) were not using tele–mental health in December 2019, only 22.07% (n=188) were not by late March or early April 2020. Only 2.11% (n=19) reported no COVID-19–related practice adjustments. Two-thirds (596/888, 67.10%) reported providing additional therapeutic services specifically to treat COVID-19–related concerns. Neuropsychologists were less likely and Psych/DL providers and SW/ML providers were more likely than expected to transition to tele–mental health (*P*<.001). Trainees saw fewer patients (*P*=.01) and worked remotely more than licensed practitioners (*P*=.03). Despite lower rates of information technology service access (*P*<.001), private practice providers reported less difficulty implementing tele–mental health than providers in other settings (*P*<.001). Overall, the majority (530/889, 59.62%) were interested in continuing to provide tele–mental health services in the future.

**Conclusions:**

The vast majority of mental health providers in this study made practice adjustments in response to COVID-19, predominantly by rapidly transitioning to tele–mental health services. Although the majority reported providing additional therapeutic services specifically to treat COVID-19–related concerns, only a small subset endorsed offering such services to medical providers. This has implications for future practical directions, as frontline workers may begin to seek mental health treatment related to the pandemic. Despite differences in tele–mental health uptake based on provider characteristics, the majority were interested in continuing to provide such services in the future. This may help to expand clinical services to those in need via tele–mental health beyond the COVID-19 pandemic.

## Introduction

In December 2019, SARS-CoV-2—more commonly referred to as COVID-19 [1]—was identified in Wuhan, China. The World Health Organization formally declared COVID-19 a global pandemic on March 11, 2020, with approximately 6.8 million cases and over 192,000 virus-related deaths in the United States as of September 23, 2020 [2,3]. Unsurprisingly, there have been calls to understand COVID-19’s psychological impact and how providers are responding [4,5].

In a recent large-scale study conducted in China, the majority of respondents endorsed moderate or severe psychological impact (eg, increased depressive symptoms, anxiety, and stress) related to COVID-19 [6]. In the United States, nearly half of respondents in a nationally representative survey endorsed anxiety about contracting COVID-19, and 40% worried about serious illness or death [7]. These findings are consistent with the psychiatric and emotional sequelae of prior pandemics, including severe acute respiratory syndrome in 2002-2003 [8,9], H1N1 influenza in 2009-2010 [10,11], and Ebola in 2013-2016 [12,13]. Evidence from these and other pandemics has indicated that longer quarantine duration is associated with higher levels of psychological distress, including depression, irritability, and posttraumatic stress symptoms [14]. Notably, adverse mental health symptoms long surpassed physical symptoms during prior pandemics [15-17]. Consistent with recommendations from prior pandemics [18,19], guidelines in countries such as China and Singapore have emphasized using tele-based platforms to understand psychological impacts, disseminate accurate health information, and provide counseling services to treat COVID-19–related distress, particularly to at-risk populations such as health care workers [20-22].

Tele–mental health services (eg, via video or phone) have become more common in recent years (2% in a 2007 review [23] to around 20% recently [24,25]), offering a potential avenue for US practitioners to continue providing mental health services remotely during quarantine. Although practitioners largely agree that tele–mental health is promising [26] and effective, there remains apprehension that it is not as effective as in-person services [24,27], despite research indicating comparable effectiveness [28,29] and patient satisfaction [30,31]. Another perceived barrier is the perception of inadequate tele–mental health education and training [23,27,32,33]. Despite concerns, it is likely that more mental health practitioners may turn to tele–mental health to provide clinical services during the COVID-19 pandemic, particularly given expanded reimbursement for such services [34].

It is likely that adoption and implementation of tele–mental health may be easier for some mental health practitioners than others based on characteristics such as provider career stage, services and treatments offered, or provider setting. For example, prior studies have found that trainees and early career psychologists were less confident about implementing tele–mental health than experienced providers [32], that mental health practitioners providing testing and evaluation services used tele–mental health at a lower rate than those providing other services [25], and that providers working in Veterans Affairs (VA) and private practices were more likely to use tele–mental health than those in other settings [25].

As of yet, there is limited information about how US mental health practitioners are adjusting their practices to respond to COVID-19. This study seeks to characterize practitioners’ immediate practical response, as well as how practice adjustments may differ across various types of providers and settings. The authors hypothesized that mental health providers overall would increase services provided via tele–mental health and that certain providers would be better able to adapt to tele–mental health services than others. The analyses were exploratory, with the intention that these findings may provide a foundation for future research examining professionals’ response to increased psychological needs during pandemics.

## Methods

### Recruitment

This study was determined to be exempt from research ethics review by the Institutional Review Board affiliated with the coauthors’ university. Eligible participants included adults (ie, 18 years or older) fluent in reading English who were currently working in a behavioral or mental health field. Participants were recruited via a Qualtrics survey link disseminated to relevant professional listservs (eg, American Psychological Association, National Academy of Neuropsychology, state psychology boards), departmental listservs, mental health practitioner colleagues, and social media platforms such as Facebook. The recruitment email included a request for participants to forward the email to colleagues if willing (ie, snowball sampling). All questions were optional, and participants were informed that they could discontinue participation at any time. Eligible individuals consented to participate by submitting their responses.

### Data Collection

Online survey data were collected from March 30, 2020, to April 10, 2020. In the Qualtrics survey (see Multimedia Appendix 1), participants were asked to provide information about their demographics, patient populations, practice adjustments in response to COVID-19, perceptions of their employer’s response, and their emotional response to and perceptions about the COVID-19 pandemic. For some questions, participants were asked about their practices months before the pandemic (ie, December 2019), directly before the pandemic (ie, late February 2020), and “currently” during the pandemic (ie, whenever they completed their survey between late March and early April 2020). Of the 1220 individuals who initiated the survey, the final sample consisted of 903 participants. Data were excluded based on the following criteria: completion of less than 66% of the survey (ie, did not provide information on variables of interest in this study; n=306); younger than 18 years (n=1); not currently working in the behavioral or mental health field (eg, gym owner, retired; n=4); and responding from outside the United States (n=6), given the extremely small number and the aim to examine practices within the specific US sociopolitical context. Compared to those who completed less than 66% of the survey, those in the final sample were on average younger (t_1129_=3.53, *P*<.001); more likely to be a neuropsychologist (n=991, χ^2^_1_=9.98, *P*=.002); and less likely to be unemployed (n=991, χ^2^_1_=12.42, *P*<.001), a bachelor’s-level provider (n=991, χ^2^_1_=16.46, *P*<.001), support staff (n=991, χ^2^_1_=14.11, *P*<.001), a different type of provider (n=991, χ^2^_1_=14.20, *P*<.001), and to be employed at a law firm (n=989, χ^2^_1_=10.38, *P*=.001).

### Data Preparation

Fewer than 5% of data were missing for each variable of interest, with a few exceptions: number of patients seen remotely in December 2019 (126/903, 13.95%) and February 2020 (133/903, 14.73%), number of patients seen currently in person (75/903, 8.31%) and remotely (51/903, 5.65%), and percent of the week spent working remotely (105/903, 11.63%). Missing data were addressed using pairwise deletion. There were 12 respondents who identified as marriage and family therapists that were recoded as therapists or counselors due to the small number (master’s-level therapist or counselor: n=10, doctoral-level therapist or counselor: n=2). A medical provider category was created to encompass nonpsychiatrist physicians, psychiatric nurse practitioners or physician assistants, and registered nurses. When there was a discrepancy between respondents’ reported highest education level and reported provider type (eg, individuals with a master’s degree who self-identified as a psychologist or doctoral-level therapist or counselor, or individuals with a bachelor’s degree who self-identified as a master’s-level therapist or counselor), provider type was recoded to reflect education level (n=7) so that, for instance, individuals with a master’s degree would be described as a master’s-level provider and not a doctoral-level provider.

### Data Analysis

Analyses were conducted in SPSS Version 26 (IBM Corp) and Stata Version 14.2 (StataCorp). Outcome variables were compared across three sets of predictors: provider level (trainee vs licensed practitioner [LP]), provider type (social worker or master’s-level provider vs psychologist or doctoral-level provider vs neuropsychologist), and setting (academic medical center [AMC] vs private practice vs VA vs community mental health [CMH] setting). The trainee category comprised graduate-level practicum students, predoctoral interns, and postdoctoral fellows. Board-certified practitioners were combined with LPs (including resident physicians) because the authors did not have specific hypotheses associated with this distinction. Social workers/master’s-level (SW/ML) providers, psychologists/doctoral-level (Psych/DL) providers, and neuropsychologists were compared because these three groups comprised the majority of the sample. The same justification was employed for comparing the four previously mentioned settings.

Chi-square tests with Bonferroni corrections (for 11 comparisons, *P*<.001) were used to compare across groups on binary variables (yes=1), including whether participants worked in a setting with easy access to information technology (IT) staff and services; whether they were *not* implementing tele–mental health in December 2019, late February 2020, and currently; and whether they endorsed making various practice adjustments. Practice adjustments were as follows: not applicable (N/A), no change in practice; cancelling patient appointments; rescheduling or postponing patient appointments; using tele–mental health or virtual appointments instead of in-person appointments; restricting the types of patients scheduled for appointments (eg, by age, medical comorbidities); or other adjustment to practice. Over 5% of the sample specified using precautionary measures (eg, personal protective equipment, social distancing) as an “other” practice adjustment; as such, this was added as a category. Selecting “N/A, no change in practice” was mutually exclusive with other practice adjustments. Otherwise, practice adjustments were not mutually exclusive. Standardized residuals were examined to assess which groups significantly contributed (*z*>|1.96|) to overall chi-square differences.

One-way analyses of covariance (ANCOVAs) with Bonferroni-corrected post-hoc tests were used to compare continuous variables across groups. Continuous variables included the number of in-person, remote, and total weekly patient visits during late February 2020 and currently (ie, late March or early April 2020); the percent of time per week currently working remotely; difficulty of tele–mental health implementation (1=*easy or not at all difficult* to 5=*very difficult*); the extent to which respondents thought that their institution, employer, or practice offered adequate information and training about providing tele–mental health (1=*strongly disagree* to 5=*strongly agree*); and the likelihood of continuing to provide tele–mental health in the future (1=*very unlikely* to 5=*very likely*). Percent of time working remotely was only calculated for those who reported >0% (n=785). Respondent age was included in ANCOVA analyses as a covariate because it was significantly correlated with all continuous outcome variables except for percent of time working remotely. For each predictor variable, there were significant differences among groups in the number of patients seen in December 2019. These were considered baseline differences, so the relevant number of December 2019 patients (total, in-person, or remote) was included as a covariate when outcomes involved the number of patients seen weekly in late February 2020 or currently. As such, group differences in these analyses can be understood as differences related to COVID-19. Square root transformations were conducted on continuous variables to address concerns with normality and homogeneity of variance, as well as to reduce outliers. *F* statistics and *P* values were derived using analyses with square root transformed variables. The original, untransformed data were reported descriptively (ie, estimated marginal means [EMMs], SEs) for ease of interpretation. EMMs represent means adjusted for covariates included in the models; as such, EMMs may differ from raw means.

## Results

### Overall Sample

The 903 participants were recruited from listservs (n=362, 40.13%), personal emails (n=291, 32.26%), social media (n=239, 26.50%), or a combination thereof (n=10, 1.11%). The majority of the sample identified as heterosexual, White, non-Hispanic, and/or cisgender women (see Table 1). Respondents were predominantly LPs, with a smaller subset of trainees (see Table 2). Of nontrainees, most were SW/ML providers, Psych/DL providers, or neuropsychologists.

**Table 1:** Demographic characteristics of the full sample (N=903).^a^

Characteristic			Participants
Age (years), mean (SD)			39.50 (11.50)
**Gender, n (%)**			
	Man	149 (16.50)	
	Woman	749 (82.95)	
	Transgender man	2 (0.22)	
	Genderqueer/nonconforming	3 (0.33)	
**Race, n (%)**			
	American Indian/Alaska Native	1 (0.11)	
	Asian/Asian American	29 (3.22)	
	Black/African American	29 (3.22)	
	Hispanic/Latinx	33 (3.67)	
	White	781 (86.78)	
	Multiracial	25 (2.78)	
	Different racial identity (ie, Arab, Jewish, Mestiza)	2 (0.22)	
**Sexual orientation, n (%)**			
	Bisexual	57 (6.34)	
	Gay	24 (2.67)	
	Heterosexual	762 (84.39)	
	Lesbian	23 (2.56)	
	Queer	20 (2.22)	
	Different sexual orientation (ie, asexual, fluid, pansexual, questioning)	13 (1.44)	
**Region, n (%)**			
	Midwest	175 (19.44)	
	Northeast	129 (14.33)	
	South	425 (47.22)	
	West	171 (19.00)	
**Work status, n (%)**			
	Full-time	671 (74.31)	
	Part-time	71 (7.86)	
	Trainee	155 (17.17)	
	Not currently employed (N/A^b^)	1 (0.11)	
	Other (ie, as needed, independent contractor, self-employed)	5 (0.55)	

^a^The number of respondents who did not provide information about demographic characteristics were as follows: gender (n=1), race (n=3), sexual orientation (n=4), and region (n=3).

^b^N/A: not applicable.

**Table 2:** Professional characteristics of the full sample (N=903).^a^

Characteristic		Participants, n (%)
**Provider type**		
	Bachelor’s-level therapist/counselor	10 (1.11)
	Social worker/master’s-level therapist/counselor	153 (16.94)
	Psychologist/doctoral-level therapist/counselor	367 (40.64)
	Neuropsychologist	144 (15.95)
	Trainee (ie, graduate-level practicum student, predoctoral intern, postdoctoral fellow)	155 (17.17)
	Psychiatrist	23 (2.55)
	Other medical provider (eg, other physician, psychiatric nurse practitioner/physician assistant)	12 (1.33)
	Support staff (eg, case manager, medical assistant, psychometrist)	34 (3.77)
	Other (eg, mental health specialist, peer recovery, research project manager)	5 (0.55)
**Provider level**		
	Graduate-level practicum student	58 (6.42)
	Predoctoral intern	38 (4.25)
	Postdoctoral fellow	59 (6.59)
	Unlicensed practitioner	38 (4.25)
	Licensed practitioner	551 (61.56)
	Licensed practitioner and board-certified in specialty area	117 (13.07)
	Not applicable (eg, support staff)	34 (3.80)
**Current practice setting**		
	Private practice	196 (21.73)
	Academic medical center	172 (19.07)
	Veterans hospital or military hospital/clinic (VA^b^)	90 (9.97)
	Community mental health setting	70 (7.76)
	Psychiatric hospital or facility	50 (5.54)
	General hospital	46 (5.10)
	Rehabilitation hospital or setting	35 (3.88)
	University counseling center	23 (2.55)
	Department/graduate training clinic	20 (2.22)
	Outpatient clinic	15 (1.66)
	School	9 (1.00)
	Primary care	7 (0.78)
	Prison	5 (0.55)
	Other (eg, cancer center, employee assistance program, nonprofit organization, intensive outpatient/partial hospitalization program)	16 (1.77)
	Multiple practice settings	148 (16.41)
**Age specialty**		
	Pediatric only (ie, younger than 18 years)	85 (9.42)
	Adults only (ie, 18 years and older)	472 (52.33)
	Lifespan (ie, pediatrics and adults)	345 (38.25)

^a^The number of respondents who did not provide information about professional characteristics were as follows: provider level (n=8), practice setting (n=1), and age specialty (n=1).

^b^VA: Veterans Affairs.

The majority of the sample reported at least one practice adjustment (see Table 3), most commonly using tele–mental health rather than in-person appointments. Of the 903 respondents, only 2.11% (n=19) reported not changing their practice. Respondents saw similar numbers of patients weekly in December 2019 (mean 18.00, SD 13.25) and February 2020 (mean 17.68, SD 13.26), then saw fewer patients weekly in late March or early April 2020 (mean 14.39, SD 14.41) compared to the two prior time points (t_876_=10.41, *P*<.001 and t_876_=10.30, *P*<.001, respectively). In December 2019 (t_775_=36.99, *P*<.001) and February 2020 (t_763_=32.31, *P*<.001), respondents saw more patients in-person (December: mean 17.05, SD 12.00; February: mean 16.38, SD 12.02) than remotely (December: mean 1.11, SD 4.66; February: mean 1.62, SD 5.51). In contrast, the opposite was true currently (in-person: mean 4.92 SD 9.01; remote: mean 10.09, SD 10.75; t_799_=11.86, *P*<.001). More respondents reported using tele–mental health currently than in December 2019 or February 2020. Respondents working remotely did so for 79.05% of the week, on average. The majority reported working in a setting with easy access to IT staff and services. Over half (474/859, 55.18%) *somewhat* or *strongly agreed* that their employer offered adequate tele–mental health information and training. Almost half (329/684, 48.10%) of those implementing tele–mental health rated it as *somewhat* or *very difficult*. Over half (530/889, 59.62%) were *somewhat* or *very likely* to continue providing tele–mental health services in the future.

Of the 888 respondents, approximately two-thirds (n=596, 67.10%) reported providing additional therapeutic services specifically to treat COVID-19–related concerns (results not shown but available upon request). The most common additional services included providing individual therapy to support new and current patients (n=420, 47.30%), resources (eg, pamphlets; n=256, 28.83%), crisis care (n=158, 17.79%), and nonclinical support groups (eg, social media page; n=157, 17.68%). Smaller percentages reported providing individual (n=127, 14.30%), family (n=13, 1.46%), or group (n=59, 6.64%) therapy specifically to medical providers to support them during COVID-19.

**Table 3:** Descriptive statistics of practice adjustments, patients seen, and tele–mental health factors during COVID-19 for the full sample (N=903).

Variables			Participants
**Practice adjustments, n (%)**			
	Tele–mental health/virtual appts^a^ (vs in-person)		729 (80.82)
	Rescheduling/postponing appts		435 (48.23)
	Cancelling appts		240 (26.61)
	Restrictions on appts (eg, by patient age, medical comorbidity, recent travel)		155 (17.18)
	Precautionary measures (eg, personal protective equipment, social distancing)		53 (5.88)
	Other adjustment (eg, expanding therapeutic services, education/training-related restrictions)		38 (4.21)
	N/A^b^ (no change in practice)		19 (2.11)
**Patients seen weekly (Dec 2019)**			
	**In-person**		
		Mean (SD)	17.05 (12.00)
		Range	0-50
	**Remote/tele–mental health**		
		Mean (SD)	1.11 (4.66)
		Range	0-50
	**Total**		
		Mean (SD)	18.00 (13.25)
		Range	0-100
**Patients seen weekly (Feb 2020)**			
	**In-person**		
		Mean (SD)	16.38 (12.02)
		Range	0-50
	**Remote/tele–mental health**		
		Mean (SD)	1.62 (5.51)
		Range	0-50
	**Total**		
		Mean (SD)	17.68 (13.26)
		Range	0-100
**Patients seen weekly (current)**			
	**In-person**		
		Mean (SD)	4.92 (9.01)
		Range	0-50
	**Remote/tele–mental health**		
		Mean (SD)	10.09 (10.75)
		Range	0-50
	**Total**		
		Mean (SD)	14.39 (14.41)
		Range	0-85
**Percent of week working remotely**			
	Mean (SD)		79.05 (32.01)
	Range		1-100
**Tele–mental health**			
	Reported not implementing tele–mental health in Dec 2019^c^, n (%)		625 (80.44)
	Reported not implementing tele–mental health in late Feb 2020^d^, n (%)		580 (75.32)
	Reported not implementing tele–mental health currently, n (%)		188 (22.07)
	Reported easy access to IT^e^ services, n (%)		657 (72.84)
	**Perceived adequacy of tele–mental health training^f^**		
		Mean (SD)	3.46 (1.32)
		Range	1-5
	**Difficulty with tele–mental health implementation^g^**		
		Mean (SD)	3.07 (1.20)
		Range	1-5
	**Likelihood of continuing to provide tele–mental health services^h^**		
		Mean (SD)	3.57 (1.36)
		Range	1-5

^a^appt: appointment.

^b^N/A: not applicable.

^c^The valid percent is presented in the table; including missingness (14.0%), the raw value was 69.21%.

^d^The valid percent is presented in the table; including missingness (14.7%), the raw value was 64.23%.

^e^IT: information technology.

^f^Five-point Likert scale (1=*strongly disagree* to 5=*strongly agree*).

^g^Five-point Likert scale (1=*easy* or *not at all difficult* to 5=*very difficult*).

^h^Five-point Likert scale (1=*very unlikely* to 5=*very likely*).

### Differences by Provider Level

Trainees (55/155, 35.48%) were more likely to cancel appointments than LPs (161/668, 24.14%; n=822, χ^2^_1_=8.36, *P*=.004). Trainees saw fewer patients weekly than LPs in February 2020 (trainee: EMM=10.22, LP: EMM=19.49; *F*_1,805_=3.92, *P*=.048, η_p_^2^=0.005) and currently (trainee: EMM=13.38, LP: EMM=14.73; *F*_1,797_=6.41, *P*=.01, η_p_^2^=0.008; Table 4).

**Table 4:** Results of chi-squares for practice adjustments and analyses of covariance for patients seen and tele–mental health factors during COVID-19 by provider level.

Variables		Trainee^a^ (n=155)	Licensed practitioner (n=668)	*P* value
**Practice adjustments, n (%)**				
	Tele–mental health/virtual appts^b^ (vs in-person)	127 (81.94)	546 (81.86)	.98^c^
	Rescheduling/postponing appts	77 (49.68)	320 (47.98)	.70^c^
	Cancelling appts	55 (35.48)	161 (24.14)	.004^c^
	Restrictions on appts (eg, by patient age, medical comorbidity, recent travel)	22 (14.19)	118 (17.69)	.30^c^
	Precautionary measures (eg, personal protective equipment, social distancing)	8 (5.16)	40 (6.00)	.69^c^
	Other adjustment (eg, expanding therapeutic services, education/training-related restrictions)	6 (3.87)	29 (4.35)	.79^c^
	N/A^d^ (no change in practice)	3 (1.94)	11 (1.65)	.80^c^
**Patients seen weekly (Dec 2019), EMM^e^ (SE)**				
	In-person	11.36 (0.99)	18.49 (0.45)	<.001
	Remote/tele–mental health	0.55 (0.41)	1.23 (0.19)	.02
	Total	11.85 (1.07)	19.53 (0.49)	<.001
**Patients seen weekly (Feb 2020), EMM (SE)**				
	In-person	15.95 (0.43)	16.62 (0.19)	.04
	Remote/tele–mental health	1.49 (0.31)	1.47 (0.14)	.62
	Total	17.23 (0.43)	17.87 (0.19)	.048
**Patients seen weekly (current), EMM (SE)**				
	In-person	3.98 (0.75)	5.00 (0.33)	.09
	Remote/tele–mental health	6.77 (0.91)	10.87 (0.42)	<.001
	Total	13.38 (0.87)	14.73 (0.39)	.01
Percent of week working remotely, EMM (SE)		86.26 (2.87)	77.84 (1.46)	.03
**Tele–mental health**				
	Reported not implementing tele–mental health in Dec 2019, n (%)	129 (94.16)	438 (76.04)	<.001^c^
	Reported not implementing tele–mental health in late Feb 2020, n (%)	115 (84.56)	411 (72.23)	.003^c^
	Reported not implementing tele–mental health currently, n (%)	40 (27.03)	115 (17.24)	.02^c^
	Easy access to IT^f^ services, n (%)	123 (79.35)	475 (71.21)	.04^c^
	Perceived adequacy of tele–mental health training, EMM (SE)	3.50 (0.12)	3.48 (0.05)	.71
	Difficulty with tele–mental health implementation, EMM (SE)	3.28 (0.12)	3.00 (0.05)	.04
	Likelihood of continuing to provide tele–mental health services, EMM (SE)	3.47 (0.12)	3.63 (0.05)	.34

^a^Trainee includes graduate-level practicum students, predoctoral interns, and postdoctoral fellows.

^b^appt: appointment.

^c^Based on Bonferroni adjustment for chi-square tests.

^d^N/A: not applicable.

^e^EMM: estimated marginal mean.

^f^IT: information technology.

Trainees (86.26%) reported working remotely for a larger percentage of the week than LPs (77.84%; *F*_1,626_=5.00, *P*=.03, η_p_^2^=0.008). In both December 2019 (n=713, χ^2^_1_=22.31, *P*<.001) and February 2020 (n=705, χ^2^_1_=8.81, *P*=.003), trainees (December: 129/155, 94.16%; February: 115/155, 84.56%) were more likely than LPs (December: 438/668, 76.04%; February: 411/668, 72.23%) to *not* use tele–mental health. Of those using tele–mental health, trainees (EMM=3.28) reported having more implementation difficulty than LPs (EMM=3.00; *F*_1,641_=4.13, *P*=.04, η_p_^2^=0.006).

### Differences by Provider Type

Psych/DL providers (321/367, 87.47%) were more likely and neuropsychologists (94/144, 65.73%) were less likely than expected to use tele–mental health or virtual instead of in-person appointments (n=663, χ^2^_2_=36.43, *P*<.001). SW/ML providers (44/153, 28.76%) were less likely and neuropsychologists (115/144, 80.42%) were more likely than expected to reschedule or postpone appointments (n=663, χ^2^_2_=85.37, *P*<.001). SW/ML providers (24/153, 15.69%) were less likely and neuropsychologists (62/144, 43.36%) were more likely than expected to cancel appointments (n=663, χ^2^_2_=36.28, *P*<.001; Table 5).

**Table 5:** Results of chi-squares for practice adjustments and analyses of covariance for patients seen and tele–mental health factors during COVID-19 by provider type.

Variables		(1) Social workers/master’s providers (n=153)	(2) Psychologists/doctoral providers (n=367)	(3) Neuropsychologists (n=144)	*P* value
**Practice adjustments, n (%)**					
	Tele–mental health/virtual appts^a^ (vs in-person)	133 (86.93)	321 (87.47)	94 (65.73)	<.001^b^
	Rescheduling/postponing appts	44 (28.76)	161 (43.90)	115 (80.42)	<.001^b^
	Cancelling appts	24 (15.69)	77 (20.98)	62 (43.36)	<.001^b^
	Restrictions on appts (eg, by patient age, medical comorbidity, recent travel)	21 (13.73)	55 (14.91)	37 (25.87)	.006^b^
	Precautionary measures (eg, personal protective equipment, social distancing)	14 (9.15)	18 (4.88)	5 (3.42)	.07^b^
	Other adjustment (eg, expanding therapeutic services, education/training-related restrictions)	6 (3.92)	17 (4.61)	7 (4.79)	.91^b^
	N/A^c^ (no change in practice)	2 (1.31)	6 (1.63)	3 (2.05)	.87^b^
**Patients seen weekly (Dec 2019), EMM^d^ (SE)**					
	In-person	22.09 (0.86)	19.64 (0.56)	9.89 (0.89)	<.001^e,f^
	Remote/tele–mental health	1.25 (0.30)	1.08 (0.18)	0.46 (0.30)	.006^e^
	Total	23.12 (0.90)	20.61 (0.58)	10.28 (0.93)	<.001^e,f^
**Patients seen weekly (Feb 2020), EMM (SE)**					
	In-person	17.96 (0.42)	17.70 (0.27)	16.73 (0.46)	.03^f^
	Remote/tele–mental health	2.01 (0.34)	1.53 (0.21)	0.75 (0.34)	.03^e^
	Total	19.97 (0.44)	18.93 (0.28)	17.34 (0.48)	<.001^e,f^
**Patients seen weekly (current), EMM (SE)**					
	In-person	6.76 (0.72)	4.69 (0.45)	4.83 (0.77)	.04^g^
	Remote/tele–mental health	14.71 (0.84)	12.20 (0.52)	3.45 (0.85)	<.001^e,f,g^
	Total	18.66 (0.83)	15.81 (0.53)	12.07 (0.91)	<.001^e,f,g^
Percent of week working remotely, EMM (SE)		80.17 (3.06)	80.03 (1.89)	70.84 (3.22)	.06
**Tele­­–mental health**					
	Reported not implementing tele–mental health in Dec 2019, n (%)	102 (82.26)	231 (70.86)	108 (89.26)	<.001^b^
	Reported not implementing tele–mental health in late Feb 2020, n (%)	91 (73.98)	214 (65.64)	103 (88.03)	<.001^b^
	Reported not implementing tele–mental health currently, n (%)	14 (9.66)	42 (11.90)	57 (42.54)	<.001^b^
	Easy access to IT^h^ services, n (%)	102 (66.67)	253 (68.94)	111 (77.62)	.08^b^
	Perceived adequacy of tele–mental health training, EMM (SE)	3.35 (0.11)	3.59 (0.07)	3.48 (0.12)	.23
	Difficulty with tele–mental health implementation, EMM (SE)	2.99 (0.10)	2.97 (0.07)	3.26 (0.14)	.14
	Likelihood of continuing to provide tele–mental health services, EMM (SE)	3.69 (0.11)	3.70 (0.07)	3.43 (0.12)	.16

^a^appt: appointment.

^b^Based on Bonferroni adjustment for chi-square tests.

^c^N/A: not applicable.

^d^EMM: estimated marginal mean.

^e^Significant difference between 2 and 3.

^f^Significant difference between 1 and 3.

^g^Significant difference between 1 and 2.

^h^IT: information technology.

In both February 2020 (*F*_2,648_=11.20, *P*<.001, η_p_^2^=0.033) and currently (*F*_2,644_=31.15, *P*<.001, η_p_^2^=0.088), neuropsychologists (February: EMM=17.34; current: EMM=12.07) saw fewer patients weekly than Psych/DL providers (February: EMM=18.93; current: EMM=15.81), who saw fewer than SW/ML providers (February: EMM=19.97; current: EMM=18.66). In December 2019 (n=571, χ^2^_2_=19.26, *P*<.001) and February 2020 (n=566, χ^2^_2_=21.73, *P*<.001), Psych/DL providers (December: 231/367, 70.86%; February: 214/367, 65.64%) were less likely and neuropsychologists (December: 108/144, 89.26%; February: 103/144, 88.03%) were more likely than expected to *not* use tele–mental health. Currently, SW/ML providers (14/153, 9.66%) and Psych/DL providers (42/367, 11.90%) were less likely and neuropsychologists (57/144, 42.54%) were more likely than expected to *not* use tele–mental health (n=632, χ^2^_2_=70.77, *P*<.001).

### Differences by Setting

Providers in AMCs (112/172, 65.12%) were more likely and those in private practice (76/196, 38.78%) were less likely than expected to reschedule or postpone appointments (n=528, χ^2^_3_=28.05, *P*<.001). AMC providers (61/172, 35.47%) were more likely and CMH providers (9/70, 12.86%) were less likely than expected to cancel appointments (n=528, χ^2^_3_=16.40, *P*=.001; Table 6).

**Table 6:** Results of chi-squares for practice adjustments and analyses of covariance for patients seen and tele–mental health factors during COVID-19 by setting.

Variables		(1) AMC^a^ (n=172)	(2) CMH^b^ (n=70)	(3) PP^c^ (n=196)	(4) VA^d^ (n=90)	*P* value
**Practice adjustments, n (%)**						
	Tele–mental health/virtual appts^e^ (vs in-person)	152 (88.37)	63 (90.00)	169 (86.22)	78 (86.67)	.83^f^
	Rescheduling/postponing appts	112 (65.12)	29 (41.43)	76 (38.78)	48 (53.33)	<.001^f^
	Cancelling appts	61 (35.47)	9 (12.86)	44 (22.45)	20 (22.22)	.001^f^
	Restrictions on appts (eg, by patient age, medical comorbidity, recent travel)	35 (20.35)	14 (20.00)	25 (12.76)	11 (12.22)	.13^f^
	Precautionary measures (eg, personal protective equipment, social distancing)	4 (2.33)	4 (5.71)	9 (4.59)	3 (3.33)	.55^f^
	Other adjustment (eg, expanding therapeutic services, education/training-related restrictions)	6 (3.49)	1 (1.43)	8 (4.08)	6 (6.67)	.39^f^
	N/A^g^ (no change in practice)	1 (0.58)	1 (1.43)	1 (0.51)	1 (1.11)	.85^f^
**Patients seen weekly (Dec 2019), EMM^h^ (SE)**						
	In-person	15.52 (0.88)	23.71 (1.36)	17.33 (0.83)	15.99 (1.21)	<.001^i,j,k^
	Remote/tele–mental health	1.14 (0.37)	2.06 (0.60)	0.68 (0.34)	1.69 (0.51)	.02
	Total	16.41 (0.98)	25.35 (1.53)	17.96 (0.93)	17.47 (1.36)	<.001^i,j,k^
**Patients seen weekly (Feb 2020), EMM (SE)**						
	In-person	16.13 (0.38)	16.71 (0.60)	17.53 (0.36)	16.70 (0.52)	.45
	Remote/tele–mental health	1.42 (0.29)	1.86 (0.47)	1.46 (0.27)	1.60 (0.40)	.31
	Total	17.21 (0.42)	19.00 (0.67)	18.77 (0.40)	17.86 (0.58)	.16
**Patients seen weekly (current), EMM (SE)**						
	In-person	4.09 (0.61)	3.23 (0.99)	4.92 (0.57)	3.90 (0.85)	.77
	Remote/tele–mental health	8.31 (0.84)	16.10 (1.36)	13.25 (0.78)	10.28 (1.16)	<.001^i,l^
	Total	13.03 (0.76)	15.42 (1.20)	17.79 (0.71)	14.19 (1.05)	<.001^i,l^
Percent of week working remotely, EMM (SE)		84.33 (2.56)	75.07 (4.20)	81.60 (2.41)	82.05 (4.33)	.35
**Tele–mental health**						
	Reported not implementing tele–mental health in Dec 2019, n (%)	130 (87.84)	42 (75.00)	125 (70.22)	51 (65.38)	<.001^f^
	Reported not implementing tele–mental health in late Feb 2020, n (%)	123 (84.25)	43 (72.88)	115 (66.09)	41 (53.95)	<.001^f^
	Reported not implementing tele–mental health currently, n (%)	36 (21.95)	7 (10.14)	21 (10.94)	10 (11.63)	.01^f^
	Easy access to IT^m^ services, n (%)	160 (93.02)	52 (74.29)	58 (29.59)	73 (81.11)	<.001^f^
	Perceived adequacy of tele–mental health training, EMM (SE)	3.59 (0.10)	3.40 (0.16)	3.70 (0.10)	3.90 (0.13)	.11
	Difficulty with tele–mental health implementation, EMM (SE)	3.21 (0.10)	3.26 (0.15)	2.60 (0.09)	3.25 (0.13)	<.001^j,l,n^
	Likelihood of continuing to provide tele–mental health services, EMM (SE)	3.55 (0.10)	3.66 (0.16)	3.56 (0.10)	3.89 (0.14)	.33

^a^AMC: academic medical center.

^b^CMH: community mental health.

^c^PP: private practice.

^d^VA: Veterans Affairs.

^e^appt: appointment.

^f^Based on Bonferroni adjustment for chi-square tests.

^g^N/A: not applicable.

^h^EMM: estimated marginal mean.

^i^Significant difference between 1 and 2.

^j^Significant difference between 2 and 3.

^k^Significant difference between 2 and 4.

^l^Significant difference between 1 and 3.

^m^IT: information technology.

^n^Significant difference between 3 and 4.

AMC providers (EMM=13.03) were currently seeing fewer patients weekly than providers in CMH settings (EMM=15.42) and private practice (EMM=17.79; *F*_3,511_=8.63, *P*<.001, η_p_^2^=0.048). In December 2019, AMC providers (130/172, 87.84%) were more likely than expected to *not* use tele–mental health (n=460, χ^2^_3_=19.26, *P*<.001). In February 2020, AMC providers (123/172, 84.25%) were more likely and VA providers (41/90, 53.95%) were less likely than expected to *not* use tele–mental health (n=455, χ^2^_3_=25.18, *P*<.001). Providers in AMCs (160/172, 93.02%) and VAs (73/90, 81.11%) were more likely and those in private practice (58/196, 29.59%) were less likely than expected to have easy access to IT staff and services (n=528, χ^2^_3_=180.22, *P*<.001). Of providers using tele–mental health, those in private practice (EMM=2.60) reported less implementation difficulty than providers in all other settings (AMC: EMM=3.21, CMH: EMM=3.26, VA: EMM=3.25; *F*_3,438_=9.93, *P*<.001, η_p_^2^=0.064).

## Discussion

### Transition to Tele–mental Health and Group Differences

This study highlights how US mental health providers have changed their practices within the rapidly evolving context of COVID-19, during which there have been increased mental health needs [7] as well as large-scale technological availability enabling tele-adaptation of services [35]. The authors hypothesized that mental health providers overall would increase the number of services provided via tele–mental health and that certain providers would be better able to adapt to tele–mental health services than others. Exploratory results were provided to describe how this transition has differed across specific mental health service lines. These findings may inform future mental health practices and policies as the outbreak continues to evolve worldwide.

Overall, the context of COVID-19 has led to widespread change in the mental health field, with all but 2.11% (19/903) of providers in this study making practice adjustments. Unsurprisingly, the most prominent change involved a transition from in-person to remote or virtual appointments. Consistent with prior research [24,25], this study found that tele–mental health was a relatively underused resource prior to this pandemic, even through late February 2020. In line with the hypotheses, results indicated a rapid transition to tele–mental health services during the pandemic, with uptake of tele–mental health by approximately 80% of respondents by late March or early April 2020. The expediency and scope of this transition rate was striking compared to that of tele–mental health initiatives during previous US emergency situations, such as the September 11, 2001, terrorist attacks [36] and Hurricane Katrina in 2005 [37]. This may have resulted from the unique context of stay-at-home policies and the easing of logistical barriers during the pandemic, such as increased tele–mental health reimbursement [34]. In addition, approximately 55% of providers in this study perceived having adequate tele–mental health training, which was substantially higher than in previous reports (ie, 21%-28%) [27,38]. This may reflect an overall movement toward increased tele–mental health training over time or more recent training specifically in response to COVID-19.

In this study, providers’ transition to tele–mental health appeared to be more than a stopgap measure limited to the pandemic context. The majority endorsed a desire to continue implementing tele–mental health services in the future, despite more than one-quarter reporting lack of easy access to IT services and nearly half endorsing implementation difficulty. Importantly, respondents overall saw fewer patients weekly in late March or early April 2020 than prior to the pandemic. This suggests that COVID-19–related disruptions have reduced treatment capacity (at least at the beginning of the pandemic) while mental health needs have surged [6].

Consistent with the second hypothesis, transition to tele–mental health services differed by provider type. Specifically, SW/ML providers transitioned to tele–mental health services at a higher rate than both Psych/DL providers and neuropsychologists. This may be explained by varying scopes of practice. Psychologists, and particularly neuropsychologists, are more likely than SW/ML providers to conduct testing and evaluation services, which have been associated with lower tele–mental health uptake [25]. Interestingly, in this study, this differential uptake did not seem to be associated with group differences in IT service access, perceived adequacy of tele–mental health training, or ease of implementation (for those using tele–mental health). Moreover, despite differential uptake, all provider types were equally likely to want to implement tele–mental health in the future. A speculative explanation for these findings may be providers’ anticipation of future development of assessments that are more compatible with tele-based platforms.

Exploratory analyses helped to further characterize how COVID-19 may be differentially affecting mental health providers’ practices. Prior to the COVID-19 pandemic, trainees were less likely than LPs to implement tele–mental health, but by late March or early April 2020, there were no differences in tele–mental health uptake. This differential speed of transition may be due to implementation of new policies (eg, perhaps LPs were prioritized in executing new technological advances). There were few differences in specific practice adjustments between trainees and LPs, which is logical given that trainees work under the supervision and license of LPs. A difference that did emerge, however, was that trainees appeared to be “protected” during COVID-19, such that they tended to work remotely more and saw fewer patients, above and beyond baseline differences.

With regard to practice setting, providers in AMCs were more likely than expected to cancel or postpone appointments and to see fewer patients compared to providers in private practice or CMH settings. One possible explanation for this is that mental health providers in AMCs are often part of a larger system with many types of providers, so the temporary decrease in billable services may be more financially tolerable than in private practice or CMH settings. This could have decreased the incentive for AMC providers to transition to tele–mental health services instead of using temporary measures until the pandemic resolved. Notably, however, by late March or early April 2020, AMC providers were facilitating tele–mental health services at the same rate as other providers. VA providers appeared to be relatively early adopters of tele–mental health, with higher than expected tele–mental health implementation in late February 2020; this may relate to the VA’s historical focus on telepsychology [25]. Despite lower rates of easy access to IT services, private practice providers had less implementation difficulty than those in all other settings explored in this study. It is possible that the relatively high autonomy and relatively low institutional oversight in private practice allowed for easier adoption of tele–mental health.

Consistent with previous recommendations [18-20], nearly 70% of practitioners in this study endorsed providing additional therapeutic services specifically to treat patients’ COVID-19–related concerns; these services most commonly included individual therapy, resource distribution (eg, pamphlets), crisis care, and nonclinical support groups (eg, social media). Of note, only a small subset of practitioners endorsed offering additional mental health services specifically to medical providers. This is concerning given the importance of addressing the psychological impact among at-risk groups such as frontline health care workers [19]. One possibility is that medical providers may not have sought mental health treatment yet, given the recency of the pandemic relative to survey dissemination and data collection. It is probable that the need for mental health services, particularly by health care workers, will increase over time as the physical symptoms of COVID-19 eventually remit and the psychological distress likely remains [15,16].

### Implications

Encouragingly, study results indicated that mental health practitioners demonstrated the ability to transition to tele–mental health services rapidly and at relatively high rates. Lower uptake by practitioners who are more likely to provide testing and evaluation services may be mitigated by working with companies to consider tele–mental health services when developing new cognitive or psychological tests and psychometric norms. This likely presented a barrier particularly for neuropsychologists, given that over 40% were not using tele–mental health by late March or early April 2020. Another key barrier to tele–mental health implementation described in previous literature has been a lack of training or education [23,32,33]. Although a higher percentage of respondents endorsed receiving adequate tele–mental health training relative to prior studies [27,38], almost half did not feel this way. This indicates an area for improvement in graduate programs and training experiences preparing individuals for mental health fields.

Given the low percentage of mental health providers offering additional therapeutic services specifically to medical providers, it will be important to make a concerted effort to identify and develop targeted mental health treatments for individuals and groups at increased risk of psychological distress related to COVID-19. This may include frontline health care workers, individuals who became unemployed, those with personal experiences with the virus, and those in geographic hot spots.

More generally, consistent with prior work [27], results from this study indicate widespread interest in continuing tele–mental health services following the COVID-19 pandemic. This could allow for increased accessibility for individuals with historically lower access to medical or mental health services (eg, due to lack of transportation, funds, or health literacy), such as those in rural locations or with low socioeconomic status. Interdisciplinary work among providers, institutions, test development companies, legislators, and insurance companies will be necessary in this endeavor.

### Limitations and Future Directions

Despite the valuable information previously noted, this study has limitations that warrant disclosure. The sample consisted predominantly of individuals identifying as White, heterosexual, married, and/or cisgender women. Almost half were from the southern region of the United States, and many were doctoral-level providers. Although the sample reflected demographic characteristics of other large-scale surveys of neuropsychologists [39] and psychologists [40], there are limits to generalizability given that this study’s sample represents a small proportion of the approximately 1.6 million US mental health professionals (ie, psychologists, counselors, social workers, and psychiatrists) as of May 2019 [41].

Other limitations included the timing of this study, which occurred relatively early in the pandemic, and the fact that analyses did not account for differential implementation of stay-at-home orders across states. However, data were collected within a relatively short time frame (ie, 12 days from survey distribution to closure of data collection), and neither completion date nor region was consistently correlated with study variables. The survey asked respondents to compare their current workload to that of December 2019, when providers may have seen fewer patients because of the holiday season. However, this would have underestimated differences between patient volumes pre–COVID-19 and during the pandemic. Future research should track COVID-19–related practice adjustments over time, as well as providers’ perceptions of their effectiveness in hindsight.

Finally, this study focused on mental health providers’ practical responses to COVID-19. It will also be important to characterize their emotional responses, given that mental health providers tend to generally have relatively high levels of job-related stress, which can impact their desire and ability to continue providing therapeutic services [42]. Recognizing that providers do not exist in a vacuum, contextualizing this within how institutions responded to the pandemic would enable a more comprehensive characterization of mental health providers’ response during COVID-19.

Overall, in the context of the current pandemic, mental health providers were able to rapidly adjust their practice, predominantly by shifting to tele–mental health services. Despite differences in tele–mental health uptake based on provider characteristics, the majority were interested in continuing to provide such services in the future. This may offer an opportunity to expand therapeutic services to those in need even beyond the COVID-19 pandemic.

## Appendix

Multimedia Appendix 1 Survey questions.

## Abbreviations

AMC: academic medical center
ANCOVA: analysis of covariance
CMH: community mental health
EMM: estimated marginal mean
IT: information technology
LP: licensed practitioner
N/A: not applicable
Psych/DL: psychologists/doctoral-level
SW/ML: social workers/master’s-level
VA: Veteran Affairs
