# Supplementary material for: Mental Health Practitioners’ Immediate Practical Response During the COVID-19 Pandemic: Observational Questionnaire Study
Source: JMIR Ment Health. 2020 Oct 1;7(10):e21237. doi: 10.2196/21237 (PMC7546864; doi:10.2196/21237)
Supplement: Multimedia Appendix 1 [file mental_v7i9e21237_app1.docx]

Appendix

**National Survey: Mental Health Practitioner Immediate Response to COVID-19**

***Thank you for your interest and participation. In this survey, you will be asked information about your practical response to COVID-19, as well as your perceptions of your institution’s response, your emotional response, and your perceptions about COVID-19. Your involvement in this project is completely voluntary, you can discontinue participating at any time while completing the survey, and you do not have to respond to questions you prefer not to answer. All information provided will be reported on as aggregated, de-identified data.***

**The following questions will ask about your demographic information and information about patients seen in your practice.**

1. What is your age? (sliding scale with specific number shown)
2. How did you hear about this survey?
   1. Professional listserv
   2. Personal email from colleague
   3. Social media
   4. Other, please specify: ________________
3. Which of the following best describes your current gender identity?
   1. Man
   2. Woman
   3. Trans-man
   4. Trans-woman
   5. Genderqueer/gender-nonconforming
   6. Non-binary
   7. Different gender identity, please specify: ____________________
4. At the present time, how do you self-identify your sexual orientation?
   1. Heterosexual or straight
   2. Gay
   3. Lesbian
   4. Bisexual
   5. Queer
   6. Questioning
   7. Different sexual orientation, please specify: ___________________
5. Do you identify as Hispanic/Latinx?
   1. Yes
   2. No
6. What race(s) do you identify as (select all that apply)?
   1. American Indian or Alaska Native
   2. Asian or Asian American
   3. Black or African American
   4. Native Hawaiian or Other Pacific Islander
   5. White
   6. Different racial identity, please specify: ________
7. What is your highest level of education?
   1. High school or equivalent (e.g., GED)
   2. Associate’s degree
   3. Bachelor’s degree
   4. Master’s degree
   5. Doctoral degree
8. What is your approximate gross household income?
   1. Less than $20,000
   2. $20,000 – $39,999
   3. $40,000 – $59,999
   4. $60,000 – $79,999
   5. $80,000 – $99,999
   6. $100,000 – $119,999
   7. $120,000 – $139,999
   8. $140,000 – $159,999
   9. $160,000 – $179,999
   10. $180,000 – $199,999
   11. $200,000 or more
9. What is your relationship status?
   1. Single
   2. In a relationship, cohabitating
   3. In a relationship, not cohabitating
   4. Married/civil union
   5. Divorced
   6. Separated
   7. Widowed
10. How many children, if any, do you have? (dropdown with numbers)
11. In which U.S. state/district/territory are you employed? (drop down, with option for N/A I am employed outside of the United States – please specify)
12. In what type of employment are you currently engaged?
    1. Full time
    2. Part time
    3. N/A, not currently employed
    4. Other, please specify _________
13. What type of provider are you? Please select one option that fits best.
    1. Bachelor’s level therapist/counselor
    2. Social worker or master’s-level therapist/counselor
    3. Psychologist or doctoral-level therapist/counselor
    4. Neuropsychologist
    5. Marriage and Family/Couple Therapist (MFTs)
    6. Psychiatrist
    7. Other physician, please specify ______
    8. Psychiatric nurse practitioner or psychiatric physician assistant
    9. Support staff
    10. Other, please specify ________
14. If support staff selected, select the type of support staff that best fits your position.
    1. Case manager
    2. Psychometrist
    3. Medical assistant
    4. Other type of support staff, please specify ________
15. Provider level (please select one):
    1. Graduate-level practicum student
    2. Pre-doctoral intern
    3. Postdoctoral fellow
    4. Unlicensed practitioner
    5. Licensed practitioner
    6. Licensed practitioner and board-certified in specialty area
16. What is your current practice setting? Select all that apply.
    1. Academic Medical Center
    2. Community Mental Health Setting
    3. Department/Graduate Training Clinic
    4. General Hospital
    5. Law Firm
    6. Prison
    7. Private Practice
    8. Psychiatric Hospital or Facility
    9. Rehabilitation Hospital or Setting
    10. School
    11. University Counseling Center
    12. Veterans Hospital or Military Hospital/Clinic
    13. Other, please specify _______
17. What patient populations do you see in your practice (setting)? Select all that apply.
    1. Forensic
    2. Inpatient
    3. Outpatient
    4. Pre-surgical (e.g., bariatric, epilepsy, pain, movement disorders)
    5. Residential
    6. Wrap-around services
    7. Other, please specify __________
18. What patient populations do you see in your practice (age)? Select all that apply.
    1. 5 and under
    2. 6-12
    3. 13-17
    4. 18-64
    5. 65+
19. What patient populations do you see in your practice (configuration)? Select all that apply.
    1. Couples
    2. Families
    3. Groups
    4. Individuals
20. Please rank **only the top three** patient populations seen in your practice (reason for referral).
    1. Behavioral concerns
    2. Behavioral medicine/health psychology
    3. Cognitive concerns (e.g., MCI/dementia)
    4. Epilepsy
    5. Family/couples counseling
    6. Medicolegal
    7. Mood disorders or other primary psychiatric concerns (not SMI)
    8. Movement disorders
    9. Multiple sclerosis
    10. Neurodevelopmental disorders
    11. Oncology/neuro-oncology
    12. Parenting concerns
    13. Serious mental illness (e.g., schizophrenia, actively suicidal)
    14. Stroke/vascular
    15. Traumatic brain injury
    16. Other, please specify _____________
21. How many patients did you see per week ***in December 2019***? (sliding scales for each)
    1. In person
    2. Remote/telehealth
22. How many patients are you ***currently*** seeing per week (i.e., the week you are completing this survey? (sliding scales for each)
    1. In person
    2. Remote/telehealth
23. Please include any additional information which you would feel would be beneficial and/or clarify your responses above, ***related to your demographic information or patients seen in your practice***. (write-in text entry)

**The following questions will ask about your response to COVID-19 as a provider.**

1. How have you adjusted your practice with patients following the COVID-19 outbreak, if at all? Please select all that apply.
   1. N/A, no change in practice
   2. Cancelling patient appointments
   3. Rescheduling/postponing patient appointments
   4. Telehealth/virtual appointments instead of in-person appointments
   5. Restricting types of patients who are scheduled (e.g., based on age, medical comorbidity, recent travel)
   6. Other adjustment to practice, please specify: _____________
2. If “Rescheduling/postponing patient appointments” is selected, How far out are you rescheduling/postponing patients on average? Please select the best option.
   1. Two-three weeks
   2. One month
   3. Two months
   4. Three months
   5. Indefinitely
3. If “Telehealth/virtual appointments instead of in-person appointments” is selected, What type of telehealth are you using? Select all that apply.
   1. Audio
   2. Audio and video
   3. Secure email
   4. Other, please specify ______
4. If “Restricting types of patients who are scheduled (e.g., based on age, medical comorbidity, recent travel)” is selected, On what bases are you restricting patient visits? Please select all that apply)?
   1. Age (e.g., if a patient is above a certain age)
   2. Medical comorbidities
   3. Recent travel
   4. Physical symptoms (e.g., if a patient has a cough, fever, shortness of breath)
   5. Crisis care (e.g., patients who are actively suicidal)
   6. Other, please specify: _______
5. If restricting patients based on: “Age” is selected, Select the range of ages for patients you ARE continuing to see in your practice. (sliding range 0-100)
6. If restricting patients based on: “Medical comorbidities” is selected, Please select the medical comorbidities you are considering in restricting patient visits currently. Select all that apply.
   1. Cardiovascular/cerebrovascular disorders (e.g., diabetes, coronary artery disease)
   2. Lung disease (e.g., COPD)
   3. Immunocompromised/immunosuppressed (e.g., HIV, multiple sclerosis)
   4. Renal disease (e.g., chronic kidney disease)
   5. Other medical comorbidity, please specify: __________
7. If restricting patients based on: “Recent travel” is selected, Please select the recent travel criteria on which you are currently restricting patient visits. Select all that apply.
   1. Out-of-state domestic travel
   2. International travel
8. If provider type: “Neuropsychologist” is selected, For neuropsychologists only: Please select the types of evaluation services you are currently providing. Select all that apply.
   1. Not currently providing any services secondary to COVID-19-related concerns
   2. Inpatient/consults
   3. In-person, interview only
   4. In-person, interview and testing
   5. Telehealth/virtual appointment, interview only
   6. Telehealth/virtual appointment, interview and testing
9. For neuropsychologists only: If “Telehealth/virtual appointment, interview and testing” is selected, Please specify the measures you are administering remotely/via telehealth. (write-in)
10. How difficult has it been to implement telehealth/virtual appointments?
    1. Easy/not at all difficult
    2. Somewhat easy
    3. Neutral (not easy or difficult)
    4. Somewhat difficult
    5. Very difficult
    6. N/A, not implementing telehealth/virtual appointments
11. Do you work in a setting with easy access to information technology (IT) staff/services?
    1. Yes
    2. No
12. If it remains available, how likely are you to continue providing services via telehealth/virtual appointments?
    1. Very unlikely
    2. Somewhat unlikely
    3. Neutral (not likely or unlikely)
    4. Somewhat likely
    5. Very likely
13. What additional therapeutic services, if any, are you providing to existing/new patients ***related specifically to COVID-19 concerns***? Please select all that apply.
    1. N/A, no additional therapeutic services
    2. Individual therapy to support existing/new patients
    3. Individual therapy to support to medical providers specifically
    4. Family therapy to support existing/new patients
    5. Family therapy to support medical providers specifically
    6. Group therapy to support existing/new patients
    7. Group therapy to support medical providers specifically
    8. Providing resources (e.g., pamphlets) to existing/new patients
    9. Community outreach (e.g., consulting with community agencies)
    10. Non-clinical support group (e.g., social media page, virtual lunch hours, peer support pairing)
    11. Crisis care
        1. If selected, are you providing crisis care: in person, remotely/via telehealth (select all that apply)
14. If “Crisis care” is selected, Are you providing crisis care related to COVID-19 concerns (select all that apply):
    1. In person
    2. Remotely/via telehealth
15. What percentage of your week are you working at home/remotely? (sliding scale 0-100, with an option to choose “Not Applicable”)
16. Are you engaging in supervision/consultation/peer support groups with colleagues related to COVID-19 concerns?
    1. Yes
    2. No
17. Please include any additional information which you would feel would be beneficial and/or clarify your responses above, ***related to your response to COVID-19 as a provider***. (write-in text entry)

**The following questions will ask about your perceptions of your institution/employer/ practice’s response to COVID-19.**

1. My institution/employer/practice has placed the following restrictions on coming into work. Please select all that apply.
   1. N/A no restrictions
   2. Travel-related restrictions
   3. Symptom-related restrictions (e.g., if an employee has a cough, fever, shortness of breath)
   4. Age-related restrictions (e.g., if an employee is over a certain age)
   5. Restrictions based on medical comorbidities (e.g., chronic illnesses, immunocompromise)
   6. No one is allowed to come into work
   7. Other, please specify: ____________
2. If “Travel-related restrictions” is selected, Please specify travel-related restrictions on coming into work placed by your institution/employer/practice. Select all that apply.
   1. Out-of-state domestic travel
   2. International travel
3. If “Age-related restrictions (e.g., if an employee is over a certain age)” is selected, select the range of ages for employees who **ARE NOT RESTRICTED** from coming into work (sliding range 0-100)
4. Please respond regarding the degree to which you agree with each statement.

|  |  | Strongly disagree | Somewhat disagree | Neutral | Somewhat agree | Strongly agree |
| --- | --- | --- | --- | --- | --- | --- |
| a. | Social distancing (e.g., 6 feet between people) is being practiced reliably in my work environment. |  |  |  |  |  |
| b. | My institution/employer/ practice was adequately prepared to address concerns/changes arising from COVID-19. |  |  |  |  |  |
| c. | My institution/employer/ practice is responding appropriately to COVID-19. |  |  |  |  |  |
| d. | My institution/employer/ practice is providing a safe working environment during COVID-19. |  |  |  |  |  |
| e. | I am satisfied with the amount of information my institution/employer/ practice has provided me about COVID-19 and associated institutional changes. |  |  |  |  |  |
| f. | Communications received from various people in my institution/practice regarding responses to COVID-19 have been consistent. |  |  |  |  |  |
| g. | My institution/employer/ practice has provided appropriate precautionary materials (e.g., hand sanitizer, gloves, masks) in my work environment. |  |  |  |  |  |
| h. | My institution/employer/ practice has provided appropriate logistical/living provisions (e.g., pay, leave to care for a dependent). |  |  |  |  |  |
| i. | My institution/employer/ practice has provided adequate information/ training about providing telehealth. |  |  |  |  |  |
| j. | My institution/employer/ practice has provided adequate information/ training about providing crisis care remotely. |  |  |  |  |  |

1. How effective do you think the institutional measures employed by your institution/employer/practice have been overall?
   1. Very ineffective
   2. Somewhat ineffective
   3. Neutral (not effective or ineffective)
   4. Somewhat effective
   5. Very effective

1. For trainees only (e.g., participants who select that they are a graduate-level practicum student, intern, or postdoctoral fellow): Please respond regarding the degree to which you agree with each statement.

|  |  | Strongly disagree | Somewhat disagree | Neutral | Somewhat agree | Strongly agree |
| --- | --- | --- | --- | --- | --- | --- |
| a. | Information and planning to respond to COVID-19 were adequately disseminated to me by my supervisor(s). |  |  |  |  |  |
| b. | My supervisor(s) have been responsive to my questions and concerns regarding operational/training changes related to COVID-19. |  |  |  |  |  |

1. Please include any additional information which you would feel would be beneficial and/or clarify your responses above, ***related to your institution/employer/practice’s response to COVID-19***. (write-in text entry)

**The following questions will ask about your emotional response to COVID-19.**

1. Please describe your personal experience(s) related to COVID-19. Please select all that apply.
   1. N/A, I have not had a personal experience with COVID-19
   2. I had symptoms but was not tested
   3. I had symptoms and had a negative test (i.e., no virus)
   4. I had symptoms and had a positive test (i.e., virus present)
   5. I have been hospitalized related to coronavirus.
   6. Someone close to me had a positive test (i.e., virus present)
   7. Someone close to me was hospitalized related to COVID-19.
2. Please rate your level of general anxiety/distress ***associated with the COVID-19 outbreak***. (sliding scale 0-10, higher number = more anxiety/distress))
3. Please rate your level of anxiety/distress ***that you or someone you know will contract COVID-19***. (sliding scale 0-10, higher number = more anxiety/distress))
4. Please rate your level of anxiety/distress ***associated with societal impacts of COVID-19*** (e.g., related to mental health, capacity of healthcare system to accommodate affected individuals, the economy). (sliding scale 0-10, higher number = more anxiety/distress))
5. Are you a primary caregiver (e.g., of a child, parent, parent-in-law, individual with a disability)?
   1. Yes
   2. No
6. If respondent selected that they are a primary caregiver, Please rate your level of anxiety/distress related to childcare/eldercare/ adult supervision related to COVID-19. (sliding scale 0-10, higher number = more anxiety/distress)
7. To what extent do you think that your anxiety/distress related to COVID-19 affects your ability to provide services to patients?
   1. N/A, I am not currently working/providing patient services secondary to COVID-19-related concerns
   2. No impact
   3. Very small impact
   4. Somewhat small impact
   5. Somewhat large impact
   6. Very large impact
8. For trainees only (e.g., participants who select that they are a graduate-level practicum student, intern, or postdoctoral fellow): To what extent are you concerned that changes related to COVID-19 will affect your training experiences/expectations?
   1. Very unconcerned
   2. Somewhat unconcerned
   3. Neutral (not concerned or unconcerned)
   4. Somewhat concerned
   5. Very concerned
9. What coping strategies have you employed to manage/alleviate anxiety/distress associated with COVID-19? Please select all that apply.
   1. Individual therapy/counseling
   2. Group therapy/counseling
   3. Supervision
   4. Peer consultation
   5. Distraction/engaging in an enjoyable activity
   6. Spending time with loved ones
   7. Exercise
   8. Relaxation apps
   9. Avoiding the feelings
   10. Rationalizing
   11. Media/social media restrictions
   12. Alcohol
   13. Tobacco
   14. Other substance use
   15. Other strategy, please specify __________
10. For each coping strategy selected above, how effective has the selected coping strategy been in managing/alleviating your anxiety/distress related to COVID-19? (choices offered for each strategy selected in the previous question)
    1. Very ineffective
    2. Somewhat ineffective
    3. Neutral (not effective or ineffective)
    4. Somewhat effective
    5. Very effective
11. Please include any additional information which you would feel would be beneficial and/or clarify your responses above, ***related to your emotional response to COVID-19***. (write-in text entry)

**The following questions will ask about your perceptions about COVID-19.**

1. When do you think the COVID-19 outbreak will be resolved in the United States?
   1. Sooner than summer 2020
   2. By summer 2020
   3. By fall 2020
   4. After fall 2020
   5. Another time, please specify: _________
2. What is the main source of your health information about COVID-19? Please select one.
   1. Internet
   2. Television
   3. Radio
   4. Social media
   5. Friends/family members
   6. Other sources, please specify: ____________
3. How satisfied are you with the amount of health information available about COVID-19?
   1. Very unsatisfied
   2. Somewhat unsatisfied
   3. Neutral (not satisfied or unsatisfied)
   4. Somewhat satisfied
   5. Very satisfied
4. Health information provided to me about COVID-19 affected me in the following ways. Please select all that apply.
   1. N/A, health information provided to me about COVID-19 has not affected me.
   2. Led me to change my behavior in an adaptive way
   3. Led me to change my behavior in a maladaptive way
   4. Led me to worry less
   5. Led me to worry more
   6. Led me to be a better provider
   7. Led me to be a worse provider
   8. Other, please specify: ___________

- You now have the option to enter your email address.  We are planning on conducting a follow-up survey in the future, and comparing your future responses to your current responses may be of benefit.
- Your email address will be stored separately from your responses and linked using a unique identifier. No one other than the study team will have access to this information. Your email will only be used as a way to contact you for the follow-up survey, which you do not have to complete.
- Your email will not be linked to any of the information that you have already provided through this survey. All information provided previously will be reported on as aggregated, de-identified data.
- Again, providing your email address is completely optional.

Email: (write-in) ___________________
